# Predicting Emerging Themes in Rapidly Expanding COVID-19 Literature With Unsupervised Word Embeddings and Machine Learning: Evidence-Based Study

**DOI:** 10.2196/34067

**Published:** 2022-11-02

**Authors:** Ridam Pal, Harshita Chopra, Raghav Awasthi, Harsh Bandhey, Aditya Nagori, Tavpritesh Sethi

**Affiliations:** 1 Department of Computational Biology Indraprastha Institute of Information Technology Delhi New Delhi India; 2 Maharaja Surajmal Institute of Technology Guru Gobind Singh Indraprastha University New Delhi India; 3 Council of Scientific & Industrial Research-Institute of Genomics and Integrative Biology New Delhi India

**Keywords:** COVID-19, named entity recognition, unsupervised word embeddings, machine learning, natural language preprocessing

## Abstract

**Background:**

Evidence from peer-reviewed literature is the cornerstone for designing responses to global threats such as COVID-19. In massive and rapidly growing corpuses, such as COVID-19 publications, assimilating and synthesizing information is challenging. Leveraging a robust computational pipeline that evaluates multiple aspects, such as network topological features, communities, and their temporal trends, can make this process more efficient.

**Objective:**

We aimed to show that new knowledge can be captured and tracked using the temporal change in the underlying unsupervised word embeddings of the literature. Further imminent themes can be predicted using machine learning on the evolving associations between words.

**Methods:**

Frequently occurring medical entities were extracted from the abstracts of more than 150,000 COVID-19 articles published on the World Health Organization database, collected on a monthly interval starting from February 2020. Word embeddings trained on each month’s literature were used to construct networks of entities with cosine similarities as edge weights. Topological features of the subsequent month’s network were forecasted based on prior patterns, and new links were predicted using supervised machine learning. Community detection and alluvial diagrams were used to track biomedical themes that evolved over the months.

**Results:**

We found that thromboembolic complications were detected as an emerging theme as early as August 2020. A shift toward the symptoms of long COVID complications was observed during March 2021, and neurological complications gained significance in June 2021. A prospective validation of the link prediction models achieved an area under the receiver operating characteristic curve of 0.87. Predictive modeling revealed predisposing conditions, symptoms, cross-infection, and neurological complications as dominant research themes in COVID-19 publications based on the patterns observed in previous months.

**Conclusions:**

Machine learning–based prediction of emerging links can contribute toward steering research by capturing themes represented by groups of medical entities, based on patterns of semantic relationships over time.

## Introduction

The COVID-19 pandemic is a global health threat and has proven to be an enigma, with its diverse clinical presentation, controversial evidence for treatment, fast-tracked vaccine development, and unclear systemic implications. Most countries have been affected by COVID-19, with around 187 million confirmed cases over a short span and more than 4 million deaths recorded until July 13, 2021 [1]. The literature around COVID-19 is growing exponentially, with more than 150,000 COVID-19 articles vetted by the World Health Organization (WHO) [2]. Understanding evolving themes in a context, such as COVID-19, is essential as knowledge synthesis from peer-reviewed literature becomes increasingly difficult for researchers, clinicians, and policymakers alike. Methods, such as topic modeling and sentiment analysis, have been previously carried out comparing preprint with peer-reviewed literature only over a short period. Ebadi et al [3] studied the temporal patterns of sentiments and the similarity between publications from different sources over time, using document embeddings. High-level research topics like oncology, personal protective equipment, analytics, rehabilitation panic, high-risk groups, and genomics were uncovered using structural topic modeling. Although such analyses reflect an abstract overview of the broad areas of research, they do not capture the evolving context between distinct domain-specific entities. The objective of our study was to analyze and track word-level semantic similarity among biomedical entities to uncover emerging themes.

Abstracts of articles hold a substantial amount of information in the literature. Named entities within abstracts play a crucial role in deducing valuable information from large amounts of text and influencing literature trends [4]. Models pretrained on biomedical, scientific, and clinical benchmark data sets have been used to extract various clinical entities, such as diseases, symptoms, chemicals, and adverse drug reactions, from continuous text. The relative context of these entities changes over time, leading to a shift in similarity with other words [5]. Unsupervised word embeddings have previously been used to capture complex science concepts using the semantic relationship signified by cosine similarity [6].

Predicting links between “medical terms” is of high significance to understand the underlying themes within the literature and the phenomenon. Link prediction is the task of predicting the existence of links between 2 nodes in a complex network based on a set of topological features. The problem of link prediction in real-world temporal networks has been explored a lot in recent years [7], primarily in online social media networks where nodes are represented by users and edges are represented by the relationship between them. Supervised learning methods based on topological proximity measures have been vastly used to capture the shifting of links across time within networks [8,9]. Our paper aims to fill these gaps through our proposed framework, EvidenceFlow [10], an interactive web application for tracking literature trends using alluvial diagrams, projection of influential entities, and network analysis across different months. We propose for the first time the use of diachronic word embeddings, link prediction in dynamic networks of entities, and machine learning to predict emerging theme literature and make these publicly available as a web application. This paper also studies the evolution of literature based on changing cosine similarity between extracted entities in weighted temporal networks and predicts future emerging trends using link prediction.

We have primarily focused on the fast emerging COVID-19 literature to train and validate our architecture for this study. We forecasted semantic and topological proximity features of named entity pairs generated from their temporal trends in prior months. Further, we used these forecasted features to predict links between clinical entities extracted from textual data over the forecasted time interval using machine learning algorithms. Furthermore, these links were used to create a network weighted by forecasted cosine similarity for detecting communities of entities that tend to reflect on the themes of the articles published in that month. To assess the efficacy of our predictive modeling, we validated the proximity features of entity pairs forecasted from autoregressive integrated moving average (ARIMA) using mean squared error (MSE). We also evaluated the machine learning algorithm’s performance for predicting the links over a time span of 3 months.

**Figure 1:**
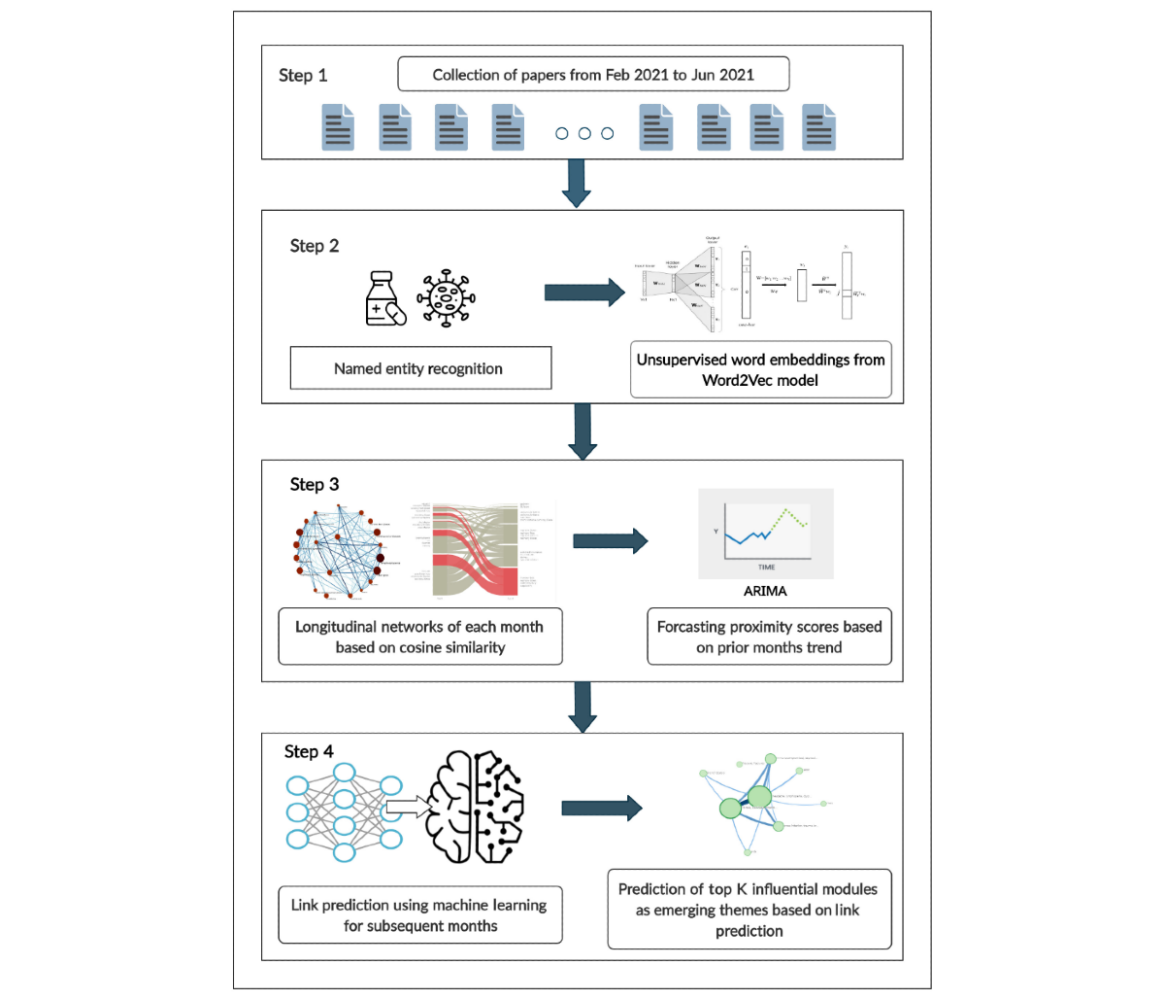
Graphical representation of the proposed framework explaining the complete workflow. The pipeline takes abstracts as inputs from which entities are extracted using named entity recognition. Embeddings are generated, which are used as features for longitudinal networks. These networks are used for visualizing the trends using alluvial diagrams, link prediction, and predicting top k influential modules for theme prediction. ARIMA: autoregressive integrated moving average.

The schematic representation of workflow has been demonstrated (Figure 1). The interactive analysis and results of emerging themes are available publicly on our web application called EvidenceFlow. The details about its working can also be found in Multimedia Appendix 1. This study proposes a framework for capturing and tracking imminent themes formed by medical entities in the temporal space based on networks constructed using word embeddings trained upon the evolving COVID-19 literature.

## Methods

### Data Set and Text Preprocessing

**Figure 2:**
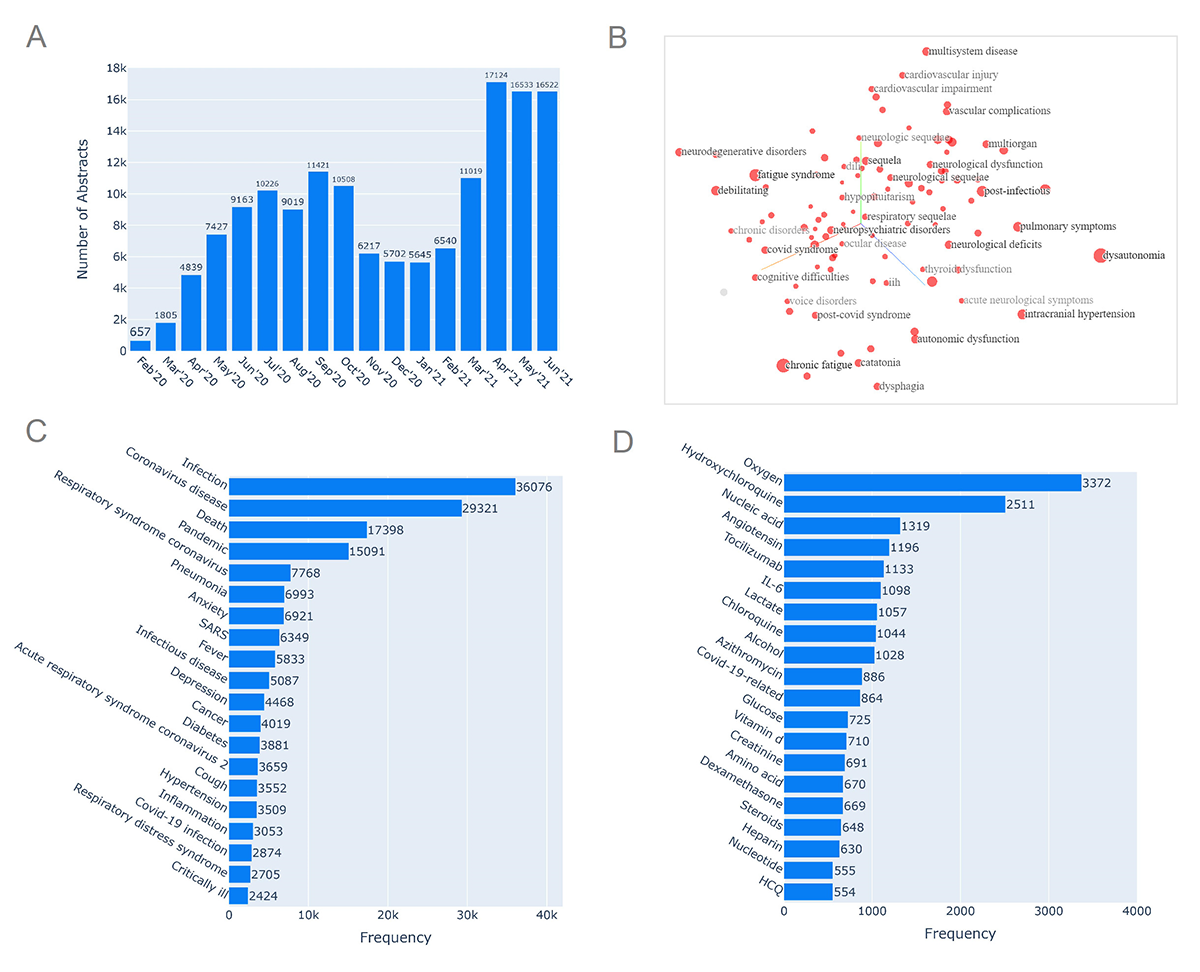
(A) Graph showing the number of articles occurring each month. The curve depicts that there has been a rampant increase in the number of articles across each month since February 2020. (B) Latent space of word embeddings of diseases visualized around the keyword “post-covid syndrome,” displaying 100 isolated points nearest to it. (C) Bar plot showing the frequency of the top diseases in the corpus of abstracts extracted using named entity recognition (NER). (D) Bar plot showing the frequency of the top chemicals in the corpus of abstracts extracted using NER. HCQ: hydroxychloroquine; IL: interleukin.

The data set was created from abstracts of approximately 150,000 COVID-19 articles published in the publicly available WHO Database [2] from February 2020 to June 2021 (Figure 2A). For every research article, the database contains the corresponding title, authors, source of publication, journal, database, language, type of publication, entry date, country, and full-text URL. We queried the database on all full-text articles in the English language, keeping the rest of the fields unfiltered. The frequency of articles concerning specific categories and keywords has been depicted in Multimedia Appendix 2. Formatting of text and removal of white spaces, punctuations, digits, and stop words were carried out on lower-case converted text using the Natural Language Toolkit (NLTK) package [11]. We list all the software and packages used in further analysis along with the corresponding versions and sources in Multimedia Appendix 3.

### Named Entity Recognition

Named entity recognition (NER) was used to extract 2 types of entities (diseases and chemicals) from the original abstracts of vetted research articles using a model pretrained on the BC5CDR corpus by SciSpacy, an open-source project for biomedical natural language processing [12]. The model identifies entities with an F1 score of 84.49% [13]. The words extracted under the category of diseases also contained symptoms, adverse effects, conditions, disorders, and syndromes. All of these are collectively referred to as diseases in the other sections. Entities were further used to create networks to study the trends through alluvial diagrams and predict links between nodes across past and upcoming months.

### Unsupervised Word Embeddings

Word embeddings were trained upon the abstracts obtained from the WHO database updated with new publications and preprints as these become available every month. A low-dimensional representation (d=100) for the words present in the corpus of abstracts was learned using the Word2Vec model with the skip-gram algorithm and a fixed window size of 5, implemented in Gensim [14-16]. Cosine distance between the word vectors of the extracted entities was calculated to analyze the dis(similarity) between entity pairs. Visualization of the word vectors was carried out using TensorFlow Embedding Projector [17] to allow interactive exploration of the relationships between diseases and chemicals. To create each month’s network of entities, separate Word2Vec models were trained to capture shifts in word similarities in the literature published over time.

### Longitudinal Entity Networks and Communities

High cosine similarity represents strong relationships between words. We used diachronic word embeddings to capture the evolving contextual similarities between various diseases and studied the evolution over time. Weighted networks were constructed using the similarity between word vectors of extracted entities as edge weights. From each month’s corpus of abstracts, top N (=100) most frequently occurring diseases were extracted, and pairs having greater than the 90th percentile of cosine similarity based on the corresponding month’s word embeddings were used to create a union set of entities across months, preserved as nodes in the temporal networks. Therefore, every month’s network had a fixed set of nodes with varying links, labeled as 0 or 1 based on the threshold of cosine similarity, and varying weights, calculated based on the evolving semantic closeness. The mentioned threshold has been chosen empirically based on experimentation; a high threshold has been selected to depict contextual similarity between 2 words present in the same latent space. For training and evaluation, a fixed set of entity pairs was created from the diseases identified in the abstracts of the papers published from February 2020 to February 2021, using the mentioned procedure. For the subsequent months, the word embedding models were trained on the respective corpora of abstracts, and the links between the fixed set of node pairs were assigned if they appeared in the vocabulary and were weighted by the cosine similarity between their word vectors. Community detection was performed over the monthly networks using the Infomap algorithm [18]. Semantic change in the word embeddings led to the formation of communities, which shifted as emerging themes over months. The importance of each node (entities) was tracked using an alluvial visualization based on PageRank values, which changed across different months [19]. Detailed steps with parameters are available in Multimedia Appendix 1.

### Time Series Forecasting of Proximity Scores

In order to predict the existence of links between nodes in the networks of subsequent months, we computed 5 neighborhood proximity scores for the network of each month. Jaccard similarity, common neighbors, preferential attachment [20], and Adamic Adar similarity [21] were used as topology-based features, and cosine similarity between the entities represented by the nodes was used as a semantic feature. These proximity scores based upon network topology were calculated using the NetworkX package [22]. Adamic Adar similarity, common neighbors, and preferential attachment values lie between 0.00 and ∞, while Jaccard similarity and cosine similarity values lie between 0.00 and 1.00. To scale the values, we normalized the former 3 scores in each network to bring them in the range of 0.00 to 1.00.

Every proximity score was modeled as a time series for each node pair, and the value was predicted for the subsequent month using the ARIMA model [23]. Stationarity of the time series was assessed using the augmented Dickey-Fuller test. A first-order autoregressive model (p=1, d=0, q=0) was used for stationary series, and nonstationary time series were passed through the random walk order of the model (p=0, d=1, q=0). For validation, proximity scores for the network at timestamp τ*+1* were predicted based on their respective past values in the networks till timestamp τ. The model’s performance was assessed by comparing the predictions with the original proximity scores in the τ*+1* time using MSE. MSE is one of the robust indicators to measure the closeness of forecast outputs to actual values in the time-series setting. To assess its sensitivity to outliers, we analyzed the distribution of errors (Multimedia Appendix 4). It was seen that the median of errors was close to zero, with minimal influence from outliers. Detailed steps with parameters are available in Multimedia Appendix 1.

### Link Prediction Between Entities

The proximity scores predicted using the ARIMA model were further used to identify the occurrence of a link between entities in network G_𝜏+1_ based on the proximity scores and links in all previous networks (G_1_, G_2_, G_3_, …, G_𝜏_), using supervised machine learning. We experimented with the proposed link prediction approach using logistic regression [24], random forest [25], support vector machine [26], AdaBoost [27], and XGBoost [28]. For training the models, 4 proximity scores (Jaccard coefficient, preferential attachment, Adamic Adar index, and common neighbors) were used as features of node pairs at each timestamp till 𝜏. For validation, the forecasted proximity scores of the network at timestamp 𝜏*+1* were used to predict links between nodes. Due to the high imbalance between the labels, the area under the receiver operating characteristic curve (AUROC) was evaluated to select the optimal threshold for binary classification. While training, validating, and testing the model, we did not use cosine similarity as a feature as it was the identifier variable for the link. Validation of the model was performed on the predicted proximity scores of April 2021 to June 2021. For logistic regression, evaluation of the key assumptions was done using the variance inflation factor for measuring the degree of multicollinearity, the Cook distance for detecting the presence of strongly influential outliers, and the scatter plot of log-odds for checking the linearity of independent variables. These tests were not satisfied for the data of most months; hence, logistic regression was not our preferred model, and we did not consider it further in the results. The Welch *t* test was performed for comparing the performance of the machine learning models, followed by Bonferroni correction [29]. The full details of the algorithm and features are available in Multimedia Appendix 1. We list the parameters set for all the models in Multimedia Appendix 5.

### Community Detection on Predicted Networks

The links between node pairs predicted by the best performing model were used to create networks weighted by cosine similarity scores predicted by the ARIMA model. The Infomap algorithm was applied on the predicted and original test network to cluster the nodes into 10 modules. The modules were compared using intersection over union (IOU) with the following formula:



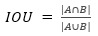



where A represents a set of nodes in the predicted ith module, i ∊ {1, 2, …, 10}, and B represents a set of nodes in the original jth module, j ∊ {1, 2, …, 10}.

## Results

Overall, 46,885 distinct diseases and 53,375 unique chemicals were identified. The top entities are shown in Figure 2C and 2D. Anxiety, depression, and hypertension were found to be present in the top 20 most discussed medical conditions in the research articles. Oxygen and hydroxychloroquine were followed by nucleic acid and angiotensin, a peptide hormone that causes vasoconstriction, among the most discussed chemicals. The latent space of word embeddings around the keyword “post-covid syndrome” visualized using a t-distributed stochastic neighbor embedding plot (Figure 2B) depicted “chronic fatigue,” “debilitating,” “neurodegenerative disorders,” and “vascular complications” among the closest medical entities in terms of cosine distance. Similar visualization for the term “mental disorders” can be found in Multimedia Appendix 6, and the top 10 most similar entities with the selected keywords “vaccine,” “comorbidity,” “adverse effects,” “social,” and “psychological” can be found in Multimedia Appendix 7.

**Figure 3:**
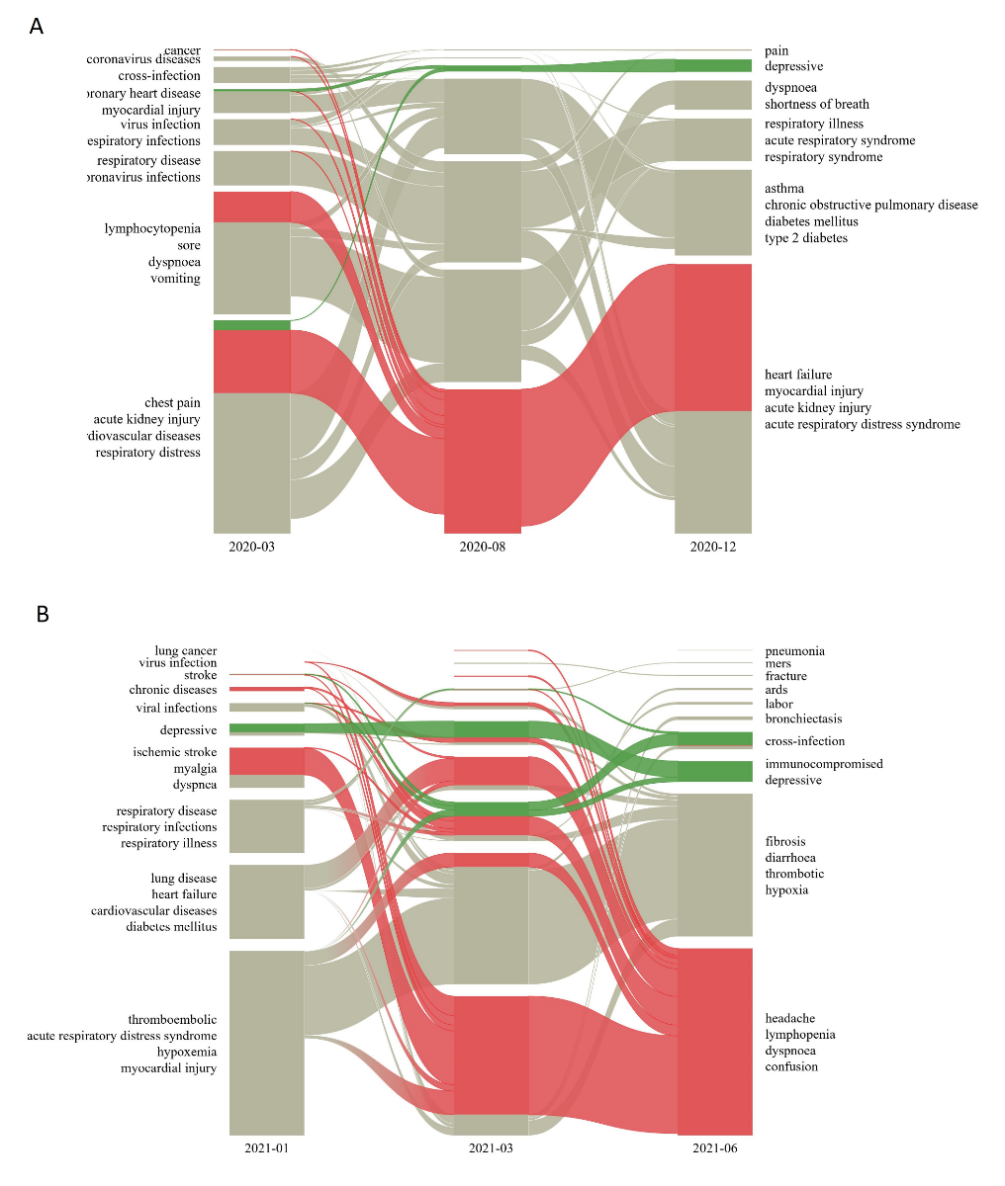
(A) Alluvial diagram for tracking the trends in 2020, from the networks of March, August, and December. (B) Alluvial diagram for monitoring the trends in 2021, from the networks of January, March, and June. The alluvial diagram eases tracing the temporal dynamics of the literature across different time intervals.

We conducted detailed inference of the alluvial diagram across different months to graphically explore the temporal trends in the literature based on dynamic and homogeneous networks of prevalent medical entities and their associated cosine similarities. Figure 3A represents the flow of themes found in the literature published in 2020. For March 2020, the dominant themes noted were chest pain, acute kidney injury, and lymphocytopenia. While there were lesser traces of “thromboembolic complications” in the literature of early months, it emerged as the most significant theme in August 2020 (Figure 3A). Myocardial injury and cardiovascular diseases surfaced as a crucial cluster of entities in December 2020. Mental health factors, such as depression, loneliness, anxiety, and burnout, gained significance in the literature of the last quarter of 2020. Figure 3B presents the flow of themes found in the literature published in 2021. While thromboembolism, hypoxemia, and myocardial infarction remained major concerns till January 2021, a significant transition toward long COVID symptoms was found as a major theme in March 2021. In June 2021, central modules, including posteffects and neurological complications, stroke, headache, and anosmia, were found to gain importance, along with newer themes around immunocompromised and chronic diseases. Cross-infection–related entities gained focus due to the second wave of COVID-19 cases in multiple countries around the world. The importance of mental health effects transitioned from lesser importance in the first quarter to more emerging and prominent links in the second quarter as highlighted in the alluvial diagram (Figure 3).

**Figure 4:**
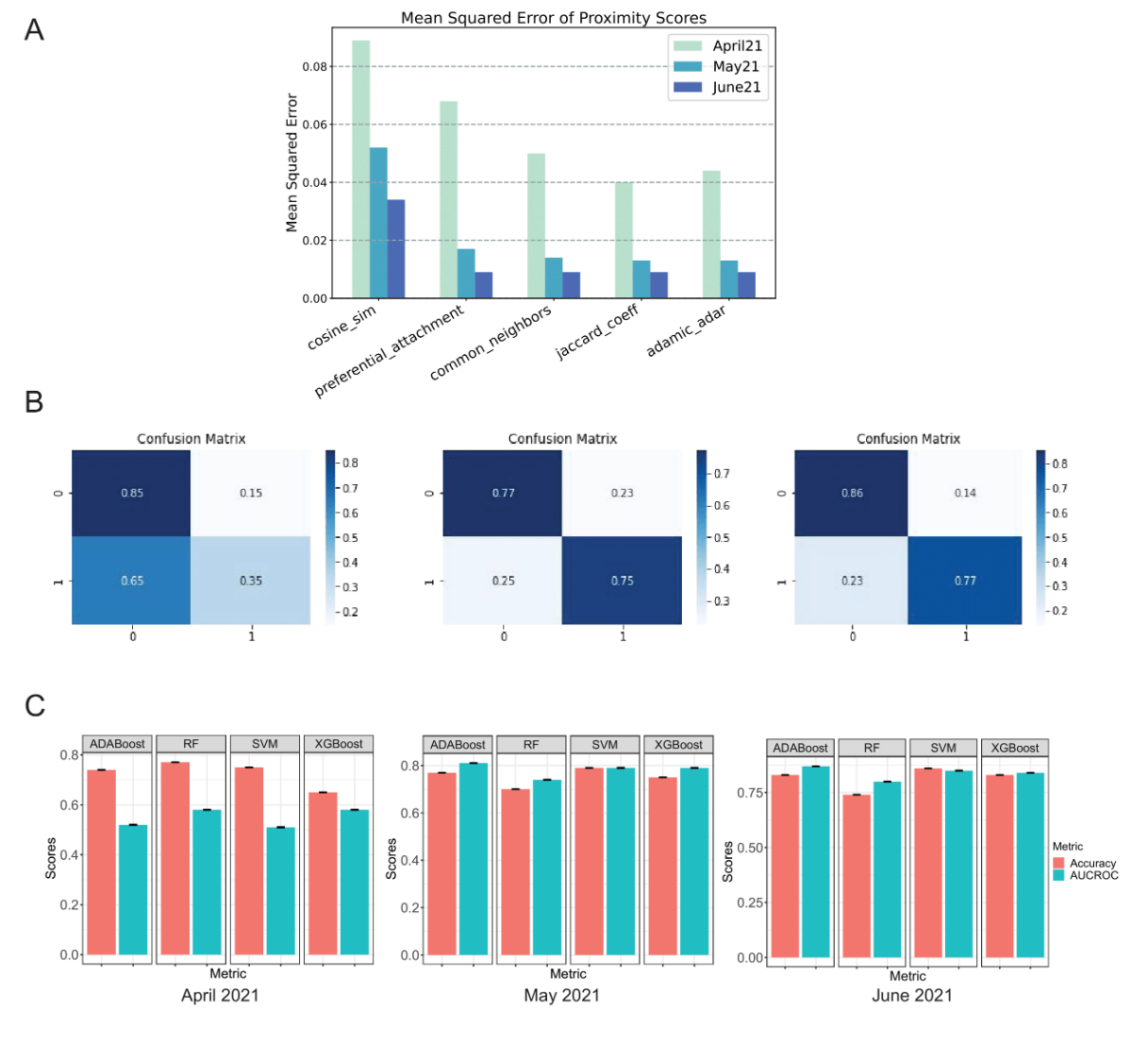
(A) Evaluation of the mean squared error (MSE) between the original and predicted proximity scores for the network of April 2021, May 2021, and June 2021. (B) Confusion matrix with normalized values of the results from the AdaBoost classifier across the months of April 2021, May 2021, and June 2021. AdaBoost has been the best performing model across all 3 months. (C) Results of link prediction between disease entities from March 2021 to June 2021, with a margin of error for 95% CIs. The mean value of metrics has been recorded by testing the models on a resampled test set. AUROC: area under the receiver operating characteristic curve; RF: random forest; SVM: support vector machine.

We further advanced the analysis of trends to predicting links between entity pairs for the upcoming months. Our proposed framework for temporal link prediction effectively forecasted 5 proximity scores, including semantic and topological measures, between node pairs by modeling the time series using the ARIMA model. The MSE in the prediction of each proximity score for April 2021, May 2021, and June 2021 is shown in Figure 4A (Multimedia Appendix 8). The associations between diseases for the successive month were predicted as links, using supervised learning based on dynamic networks belonging to the previous months. Our results showed that among the 4 classifiers (Multimedia Appendix 9), the AdaBoost model with 50 estimators and a learning rate of 0.1 classified links with a mean AUROC of 0.871 (all *P*<.001; statistically significant at a Bonferroni-corrected significance level of .02) in the test data of June 2021 (Figure 4B and 4C). Comparisons among other classifiers are shown in Multimedia Appendix 10. The predicted links weighted by forecasted cosine similarity showed a high intersection with the original modules, hence validating the proposed architecture. Multimedia Appendix 11 shows the clusters detected in the original network versus the predicted network. The ARIMA model was used for forecasting proximity scores for subsequent months based on the trends in node pair proximity measures retrieved from the prior months (February 2020 to June 2020). Our findings suggest that the themes of predisposing conditions and risk factors, and studies on cross-infection and neuropsychiatric manifestation will assume a higher centrality in the upcoming quarter of 2021 (Multimedia Appendix 12).

The intersection of nodes between the predicted and original modules was analyzed to prospectively validate the effectiveness of the proposed prediction framework. Table 1 depicts the top nodes in the different modules along with their respective IOU scores for January and June 2021. The collection of intersecting nodes has been interpreted to represent broad themes. Organ damage, like acute kidney injury and pulmonary embolism associated with COVID-19, was the most central theme in the literature from January 2021, followed by cardiovascular diseases, respiratory infections, and psychological effects. Interestingly, major themes in June 2021 shifted toward conditions related to long COVID and neurological symptoms. Headache, encephalitis, and confusion were predicted to be the central nodes, and showed a high IOU score when compared with the original network. The percentages of articles published in June 2021 mentioning entities from each module for the actual and predicted networks are presented in Multimedia Appendix 13. A subset of nodes belonging to different modules from both predicted and true networks has been presented in Multimedia Appendix 11.

Analysis of networks constructed upon chemical entities revealed the evolution of various drugs studied in the COVID-19 literature. During February 2020, the major module contained entities such as paracetamol, tofacitinib, thalidomide, vitamins, zinc, and other linked chemicals. Another relevant module included central entities, such as doxycycline, ruxolitinib, heparin, and ivermectin, which were discussed in the scientific research on the treatment and prevention of COVID-19. In contrast, our recently updated models showed the emergence of evidence for various immunosuppressive drugs, such as tacrolimus, and anti-inflammatory drugs, such as glucocorticoids and colchicine, during November 2021 (Multimedia Appendix 14). These relatively less important entities in earlier months started to become more prominent as the literature expanded. Evidence around “statins” also gained centrality over recent months. Our findings show that the proposed framework captures the dynamic changes in the importance of entities based on their evolving relationship with neighboring entities.

**Table 1:** Clusters or modules of diseases from the predicted network of January 2021 and June 2021.

Module ID	January 2021		June 2021	
	Top nodes^a^	IOU^b^	Top nodes	IOU
1	Acute kidney injury, ARDS^c^, coagulopathy, myocardial injury, pulmonary embolism	0.45	Headache, lymphopenia, dyspnea, confusion, encephalitis, nausea	0.71
2	Cardiovascular disease, diabetes mellitus, COPD^d^, hypertension	0.66	Fibrosis, coagulopathy, thrombotic, hypoxia, inflammation, delirium	0.70
3	Respiratory infection, MERS^e^, respiratory diseases	0.55	Comorbidity, asthma, COPD, hypertension, dementia, diabetes	0.64
4	Depression, insomnia, anxiety, loneliness	0.71	Traumatic, anxiety, depression, loneliness, burnout, insomnia	0.81
5	Myalgia, lymphopenia, headache, anosmia, dyspnea	0.43	Immunocompromised, chronic diseases like tuberculosis	0.33

^a^A subset of top intersecting nodes in each cluster is mentioned, which collectively signify themes.

^b^The given intersection over union (IOU) was computed between clusters of predicted and original networks of the respective months.

^c^ARDS: acute respiratory distress syndrome.

^d^COPD: chronic obstructive pulmonary disease.

^e^MERS: Middle East respiratory syndrome.

## Discussion

### Principal Findings

In this paper, we demonstrate a computational approach, EvidenceFlow, in which a user interacts with the rapidly expanding COVID-19 literature to derive and predict emerging themes. The proposed framework tracks patterns of changing semantic and topological proximity between entity pairs across months. Further, it predicts links and network communities that may emerge in future months. Hence, users can follow the papers that contribute to emerging communities of themes, for example, literature around thromboembolic complications captured as early as August 2020 and mental health factors during the end of 2020. Interacting with the clusters on the interactive interface of the EvidenceFlow model revealed that symptoms of long COVID, such as fatigue, headache, myalgia, cough, and anosmia, were forming a central cluster during March 2021. This early signal for accumulating evidence was later validated in large prospective and retrospective cohorts of COVID-19 patients [30-32]. Another way in which users can interact with EvidenceFlow is to gain an understanding of the evolution of themes going beyond current approaches such as topic modeling and sentiment tracking [3]. An example is the early finding of imminent themes around neurological complications, such as confusion, psychiatric illness, and stroke, and mental health factors, such as anxiety, depression, posttraumatic stress disorder, burnout, and insomnia, in June 2021. Our violin plot analysis (Multimedia Appendix 4) showed that despite the mean error being centered on zero, there were some outlier node pairs whose predicted associations deviated from the ground truth. The future scope of this work will involve an analysis of such associations and insights gained by an interactive analysis of such pairs on the EvidenceFlow application.

Prediction of the themes represented by rising centrality of entities can assist in the formation of promising research hypotheses. The dynamics of the literature reveal the emergence of central themes as a combination of pre-existing themes in recent times [6]. For example, the alluvial diagram (Figure 3A) demonstrated how entities from multiple modules in March 2020 merged into a major cluster of thromboembolic complications. Similarly, the flow of importance of psychological disorders over the months indicates their contemporary relevance in the COVID-19 literature and their links with other entities in the cluster. Our framework can potentially help researchers in monitoring existing themes and directing their studies based on trends and predictions.

We conducted an analysis on the trends of the PageRank centrality of selected chemical and disease entities. Statins, a class of lipid-lowering medications, were found to be gaining centrality in late 2021 as compared to earlier values (Multimedia Appendix 15). Numerous studies discussed statins for having anti-inflammatory and immunomodulatory effects that may reduce the severity of COVID-19 [33,34]. Glucocorticoids, a class of steroid hormones that reduce inflammation and suppress the immune system, also emerged as a rising entity (Multimedia Appendix 15). Depression and other mental health disorders started becoming a prominent topic of research during the middle of 2020 and gained higher importance in subsequent months (Multimedia Appendix 15). COVID-19 has also been largely discussed in the context of a thromboembolism, and our model captured its emerging evidence as a theme till late 2020. However, the trends showed that its centrality in the literature relatively decreased in 2021 (Multimedia Appendix 15). Discovering such trends from a large corpus is indeed possible using manual curation and analysis by experts. However, our EvidenceFlow pipeline provides an efficient lens to discover, track, and predict emerging trends. This framework will enable faster synthesis of evidence, which then can be validated by experts.

**Figure 5:**
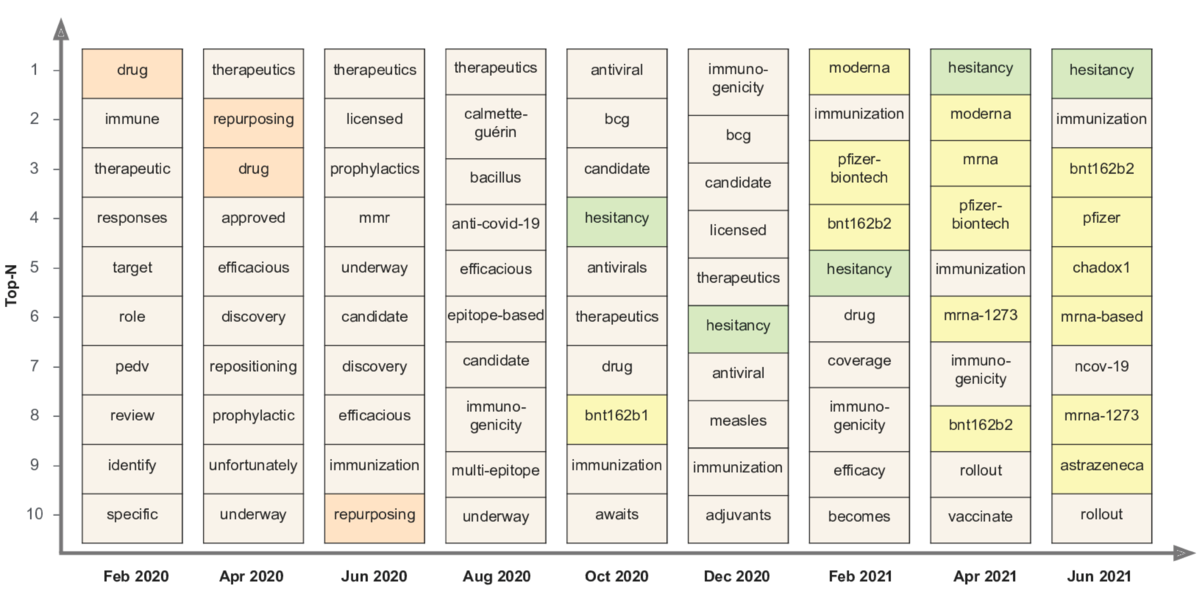
Temporal evolution of the context of the term “vaccine” across alternate months. The top 10 most similar words based on cosine similarity using monthly Word2Vec embeddings are plotted. Origin and evolution of drug repurposing in the early months, hesitancy, and vaccine candidates in the later months are highlighted.

To explore the potential of unsupervised word embeddings and changing cosine similarity among words, we analyzed the trends of terms having maximum similarity with selected keywords. For example, we analyzed the temporal shift in the context of “vaccine” over the months by finding the top 10 terms most similar to *vaccine* in the latent space of word embeddings trained on the abstracts from each month (Figure 5). From February to August 2020, research on COVID-19 vaccines was underway, and the studies revolved around “therapeutics,” “prophylactics,” “drug repurposing,” and associations with the MMR (measles-mumps-rubella) vaccine and BCG (Bacillus Calmette–Guérin) vaccine. As the clinical trials of certain vaccine candidates became prominent after August 2020, the theme of vaccine *hesitancy* emerged in October 2020 and gained higher similarity in subsequent months. Additionally, as the literature evolved in 2021, a wide range of COVID-19 vaccines, such as BNT162b1, Pfizer-BioNTech, AstraZeneca, ChAdOx1, mRNA-1273, and Moderna, were found to be majorly discussed in the context of research on vaccines. Terms, such as *immunogenicity* and *efficacy*, further suggested high association with vaccine trials and rollouts. Recently updated models showed the emergence of “booster” doses from August 2021 onwards. Such retrospective evaluation of the development of evidence from the literature over time can assist the research community in deriving detailed insights leveraging the applications of word embeddings.

### Limitations

Our study has some limitations. First, although the WHO database has been built using a detailed search strategy for COVID-19 literature, it does not explicitly report the exact purpose or accuracy of the search and decision process. The documentation [35] mentions screening done by expert reviewers and an attempt to remove duplicates, but further details are lacking. For example, the process does not clarify if redundancy across various publishers was taken care of. Further, the frequent use of the “OR” combination of keywords may have led to the inclusion of less relevant articles, while other forms of literature, such as patent applications, which can add value to the study, were not included in this database. Nonetheless, we chose the WHO COVID-19 database as it provides a large collection of articles that are updated regularly from searches of multiple bibliographic databases [2]. This, combined with curated expert-referred scientific articles, which would not be readily accessible on a custom search, was useful for building the EvidenceFlow pipeline. Future work with this framework will include potential extension to databases curated through both generic queries and expert vetting, thus facilitating targeted evidence synthesis from a variety of databases.

Further, we are currently using abstracts of research articles to extract named entities and may be missing on the details contained in the full text of the articles while training word embeddings. Therefore, future work may build upon the framework to include the full text of articles wherever available. The NER model used in our study has been reported to have achieved an F1 score of 84.49% on a benchmark data set [13]. Despite the limitations of the F1 score, such as equal weightage given to precision and recall [36,37], F1 remains one of the most widely reported performance indicators. We chose this metric in the absence of other metrics reported for this NER model. For forecasting, we used a relatively basic model (autoregressive approach), as our goal was to capture robust patterns. However, further research is possible for the use of more complex time-series approaches with higher-order difference and lags. Moreover, as the number of timestamps and data points increase, advanced architectures, such as recurrent neural network and long short-term memory [38,39], can be used for handling complex trends in the time series efficiently. Further experiments with larger networks can reveal themes that were not found with the top 100 entities. Importantly, our model is supporting the early detection of emerging trends, but it cannot capture themes on which no evidence has been accumulated.

### Conclusion

Consortia across the globe were formed for the advancement of research related to COVID-19. The global attention has led to a widespread increase in the scientific literature to study and prevent the disease from spreading, resulting in an understanding of the disease from multiple perspectives. We introduced a framework built upon COVID-19–specific literature vetted by the WHO and deployed as a dashboard called EvidenceFlow [10]. The dashboard allows the user to unravel the literature with an interactive map of embeddings based on the visualization provided by Tensorboard. It aims to track literature trends using alluvial diagrams, multilevel community detection, and projection of influential entities through network analysis across different months. This study presented how machine learning–based prediction of emerging links can contribute toward analyzing research by capturing themes represented by groups of medical entities, based on patterns of semantic relationships over time.

## Appendix

Multimedia Appendix 1 Supplementary text.

Multimedia Appendix 2 Frequency of articles belonging to specific categories in the COVID-19 literature.

Multimedia Appendix 3 List of software and packages used for our study with their sources and identifiers for the reproducibility of this study.

Multimedia Appendix 4 Distribution of errors in the prediction of proximity scores between node pairs (used as features in model training) for the month of June 2021.

Multimedia Appendix 5 Models and respective parameters used for training.

Multimedia Appendix 6 Latent space of word embeddings of diseases and chemicals visualized around the keyword “mental disorders,” displaying 100 isolated points nearest to it.

Multimedia Appendix 7 The top 10 similar entities (diseases, conditions, or chemicals) with selected keywords (“vaccine,” “comorbidity,” “adverse effects,” “social,” and “psychological”) in descending order of cosine similarity calculated using the word embeddings generated from the Word2Vec model trained on the entire corpus.

Multimedia Appendix 8 Evaluation of the mean squared error between original and predicted proximity scores for the network of April 2021, May 2021, and June 2021.

Multimedia Appendix 9 Results of temporal link prediction between entities for the months of April 2021, May 2021, and June 2021, with a margin of error for 95% confidence intervals.

Multimedia Appendix 10 Welch t test results of the performance of algorithms for the test set of June 2021.

Multimedia Appendix 11 Community detection results from the predicted and actual networks for June 2021.

Multimedia Appendix 12 Results of community detection from the predicted subsequent network based on training data till June 2021.

Multimedia Appendix 13 Percentage of abstracts of articles published in June 2021 mentioning diseases belonging to each module in the actual (A) and predicted (B) networks.

Multimedia Appendix 14 Alluvial diagram for tracking the trends of chemical entities from the networks of February 2020 to November 2021.

Multimedia Appendix 15 Temporal trends of the PageRank centrality of (A) “statins,” (B) “glucocorticoids,” (C) “depressive,” and (D) “thromboembolic”.

## Abbreviations

ARIMA: autoregressive integrated moving average
AUROC: area under the receiver operating characteristic curve
IOU: intersection over union
MSE: mean squared error
NER: named entity recognition
WHO: World Health Organization

## References

[1] Coronavirus disease (COVID-19) Weekly Epidemiological Update and Weekly Operational Update. World Health Organization.

[2] Global research on coronavirus disease (COVID-19). World Health Organization.

[3] Ebadi A, Xi P, Tremblay S, Spencer B, Pall R, Wong A (2021). Understanding the temporal evolution of COVID-19 research through machine learning and natural language processing. Scientometrics.

[4] Cho H, Lee H (2019). Biomedical named entity recognition using deep neural networks with contextual information. BMC Bioinformatics.

[5] Kutuzov A, Øvrelid L, Szymanski T, Velldal E (2018). Diachronic word embeddings and semantic shifts: a survey. Proceedings of the 27th International Conference on Computational Linguistics.

[6] Tshitoyan V, Dagdelen J, Weston L, Dunn A, Rong Z, Kononova O, Persson KA, Ceder G, Jain A (2019). Unsupervised word embeddings capture latent knowledge from materials science literature. Nature.

[7] Bu Z, Wang Y, Li H, Jiang J, Wu Z, Cao J (2019). Link prediction in temporal networks: Integrating survival analysis and game theory. Information Sciences.

[8] Özcan A, Öğüdücü ŞG (2017). Supervised temporal link prediction using time series of similarity measures.

[9] Güneş İ, Gündüz-Öğüdücü Ş, Çataltepe Z (2015). Link prediction using time series of neighborhood-based node similarity scores. Data Min Knowl Disc.

[10] What is EvidenceFlow?. EvidenceFlow.

[11] Bird S, Loper E (2004). NLTK: The Natural Language Toolkit. Proceedings of the ACL Interactive Poster and Demonstration Sessions.

[12] Neumann M, King D, Beltagy I, Ammar W (2019). ScispaCy: Fast and Robust Models for Biomedical Natural Language Processing. Proceedings of the 18th BioNLP Workshop and Shared Task.

[13] scispacy. GitHub.

[14] Ma L, Zhang Y (2015). Using Word2Vec to process big text data.

[15] Mikolov T, Sutskever I, Chen K, Corrado G, Dean J Distributed Representations of Words and Phrases and their Compositionality. Neural Information Processing Systems.

[16] Rehurek R, Sojka P Gensim–python framework for vector space modelling. NLP Centre, Faculty of Informatics, Masaryk University.

[17] Smilkov D, Thorat N, Nicholson C, Reif E, Viégas F, Wattenberg M (2016). Embedding projector: Interactive visualization and interpretation of embeddings. arXiv.

[18] Bohlin L, Edler D, Lancichinetti A, Rosvall M, Ding Y, Rousseau R, Wolfram D (2014). Community Detection and Visualization of Networks with the Map Equation Framework. Measuring Scholarly Impact.

[19] Rosvall M, Bergstrom CT (2010). Mapping change in large networks. PLoS One.

[20] Barabasi AL, Albert R (1999). Emergence of scaling in random networks. Science.

[21] Adamic LA, Adar E (2003). Friends and neighbors on the Web. Social Networks.

[22] Hagberg A, Swart P, S Chult D (2008). Exploring network structure, dynamics, and function using networkx. Office of Scientific and Technical Information.

[23] Zhang G (2003). Time series forecasting using a hybrid ARIMA and neural network model. Neurocomputing.

[24] Wright RE, Grimm LG, Yarnold PR (1995). Logistic regression. Reading and understanding multivariate statistics.

[25] Breiman L (2001). Random forests. Machine Learning.

[26] Hearst M, Dumais S, Osuna E, Platt J, Scholkopf B (1998). Support vector machines. IEEE Intell. Syst. Their Appl.

[27] Freund Y, Schapire RE (1999). A short introduction to boosting. Journal of Japanese Society for Artificial Intelligence.

[28] Chen T, He T, Benesty M, Khotilovich V, Tang Y, Cho H Xgboost: extreme gradient boosting. R package version 4–2. R Project.

[29] No authors (2015). Etymologia: Bonferroni correction. Emerg Infect Dis.

[30] Taquet M, Dercon Q, Luciano S, Geddes JR, Husain M, Harrison PJ (2021). Incidence, co-occurrence, and evolution of long-COVID features: A 6-month retrospective cohort study of 273,618 survivors of COVID-19. PLoS Med.

[31] López-León S, Wegman-Ostrosky T, Perelman C, Sepulveda R, Rebolledo P, Cuapio A, Villapol S More than 50 Long-Term Effects of COVID-19: A Systematic Review and Meta-Analysis. SSRN.

[32] Blomberg B, Mohn KG, Brokstad KA, Zhou F, Linchausen DW, Hansen B, Lartey S, Onyango TB, Kuwelker K, Sævik M, Bartsch H, Tøndel C, Kittang BR, Cox RJ, Langeland N, Bergen COVID-19 Research Group (2021). Long COVID in a prospective cohort of home-isolated patients. Nat Med.

[33] Daniels L, Ren J, Kumar K, Bui Q, Zhang J, Zhang X, Sawan M, Eisen H, Longhurst C, Messer K (2021). Relation of prior statin and anti-hypertensive use to severity of disease among patients hospitalized with COVID-19: Findings from the American Heart Association's COVID-19 Cardiovascular Disease Registry. PLoS One.

[34] Peymani P, Dehesh T, Aligolighasemabadi F, Sadeghdoust M, Kotfis K, Ahmadi M, Mehrbod P, Iranpour P, Dastghaib S, Nasimian A, Ravandi A, Kidane B, Ahmed N, Sharma P, Shojaei S, Bagheri Lankarani K, Madej A, Rezaei N, Madrakian T, Los MJ, Labouta HI, Mokarram P, Ghavami S (2021). Statins in patients with COVID-19: a retrospective cohort study in Iranian COVID-19 patients. Transl Med Commun.

[35] WHO COVID-19 Sources Search Strategy. World Health Organization.

[36] Hand D, Christen P (2017). A note on using the F-measure for evaluating record linkage algorithms. Stat Comput.

[37] Powers DMW Evaluation: from precision, recall and F-measure to ROC, informedness, markedness and correlation. arXiv.

[38] Sherstinsky A (2020). Fundamentals of recurrent neural network (RNN) and long short-term memory (LSTM) network. Physica D: Nonlinear Phenomena.

[39] Greff K, Srivastava RK, Koutnik J, Steunebrink BR, Schmidhuber J (2017). LSTM: A search space odyssey. IEEE Trans. Neural Netw. Learning Syst.

