# Supplementary material for: Predicting Emerging Themes in Rapidly Expanding COVID-19 Literature With Unsupervised Word Embeddings and Machine Learning: Evidence-Based Study
Source: J Med Internet Res. 2022 Nov 2;24(11):e34067. doi: 10.2196/34067 (PMC9629347; doi:10.2196/34067)
Supplement: Multimedia Appendix 1 [file jmir_v24i11e34067_app1.docx]

**Multimedia Appendix 1**

**EvidenceFlow.**

EvidenceFlow is an open-source interactive web application built upon COVID-19 specific literature vetted by the WHO, for tracking literature trends using alluvial diagrams, projection of influential entities, and network analysis across different months. The dashboard assists the user to understand the current and upcoming trends in the literature. The functionality of each tab has been mentioned below.

*Alluvial Diagram*: Select two or more months from the Add Month section, one by one. Once the months are selected, you can drag and change the order of these months in the Node/Month list that appears below. Click on the Create Diagram button to visualize the alluvial diagram. The alluvial diagram helps in tracking the trends in the literature between the selected months. It eases tracing the temporal dynamics of literature across different time intervals. In the Module Explorer panel on the right, there are multiple features that can help in better visualization, e.g., by painting all nodes of a selected module.

*Multi-level Network*: Select a specific month from the “Select Month” button. This illustrates the communities in the networks that are formed across entities extracted from the literature. More information about this network is given in the paper.

*Source-level Network*: Select a specific month from the “Select Month” button. This illustrates the source networks that are formed across diseases entities extracted from the literature. The link between two diseases suggests an association between two entities based on the cosine similarity.

*Embedding Projector*: Once we click on the “Embedding Projector” tab, it illustrates the latent space of the low dimensional word embeddings trained on the literature of COVID-19. The search option allows the user to query the nearest entity present across it. Isolate point allows the user to isolate N nearest points present around it.

*Emerging Trends*: This tab demonstrates the forecasted trends for the upcoming months based on the Link Prediction of entities.

*Overview*: This demonstrates the architecture of our current study. We have also attached the link to the paper in that tab.

*Extra Features*: This tab cumulates two features, “Text Summarisation” and “Word Algebra”. Text summarisation allows text summary of keywords from the abstract of literature. It highlights important points related to searched keywords from the extensive corpus of abstracts. The “Word Algebra” facilitates the linking of dimensional space based on vector algebra. It instantiates an intuition related to the vector space of the corpus for which the language model has been trained.

#

# **Longitudinal Entity Networks and Communities.**

We considered the top N(100) entities from the abstracts of papers published each month based on the frequency. Corresponding networks were constructed using the following algorithm:

1. For every month 𝜏 in T months,
   1. Top N entities extracted from the corpus of abstracts using NER.
   2. All possible pairs of entities (^N^C_2_ combinations) are created to represent node pairs in a network. The weight of the edges is valued by the Cosine Similarity between the entities generated from the Word2Vec model trained on the corpus of month 𝜏.
   3. Edges weighing more than the M^th^ percentile of the weight are preserved as links.
2. A union of node pairs (Δ = ⋃*(u,v)*) from all the months were taken to have a common set of nodes in each. Every month 𝜏 was depicted by a network G_𝜏._

**Time Series Forecasting of Proximity Scores: Parameters for ARIMA.**

The time series forecasting was performed using the ARIMA model from the statsmodels package of Python. Each pairwise association in the network can vary temporally. The time series for each association (proximity score) may have different parameters. We tested each time series for stationarity using the Augmented Dickey-Fuller test, considering stationarity for P<.05. An ARIMA approach was then taken. If the series were stationary, a first-order autoregressive model (AR) model (p,d,q=1,0,0) was fit, else a random walk order (p,d,q=0,1,0) was used.

# **Link Prediction between Entities.**

The algorithm used to predict links in the network at timestamp 𝜏+1 has been demonstrated below.

1. For each node pair *(u,v)* ∈ Δ in G_t_, t ∈ {1, 2,…,𝜏), five proximity scores were calculated based on the topological features of the graph and semantic similarity between entities.

○ Cosine Similarity, from the Word2vec model trained on the corpus of month 𝜏.

○ Jaccard Coefficient (JC)

○ Number of Common Neighbors (CN)

○ Preferential Attachment (PA)

○ Adamic-Adar Index (AA)

○ Since the range of CN, PA and AA lies between 0.00 − ∞, we normalized the respective scores in the range of 0.00 − 1.00 in each network G_t_.

1. For each proximity score, a (Δ𝜏) matrix was created where Δ represents the number of node pairs, and 𝜏 represents the number of months taken in the training set. This matrix stores the value of the proximity score for node pair *(u,v)* ∈ Δ at timestamp t.
2. For each node pair, the value of proximity score was forecasted at timestamp 𝜏+1 using the ARIMA model (p=1, d=0, q=0) if the series was stationary, else random walk order was used (p=0, d=1, q=0). The Mean Squared Error was calculated for the predicted proximity score for April 2021, May 2021, and June 2021.
3. Training of the classification model was done using four topological proximity scores as features (excluding cosine similarity as it is an identifier variable) from the networks G_1_, G_2_,…, G_𝜏._
4. Testing set features represent the four predicted proximity scores G_𝜏+1_ for all the node pairs *(u,v)* ∈ Δ.
5. Due to a high imbalance between positive and negative labels, the Receiver Operator Curve (ROC) was used to obtain an optimal threshold for the binary classification, using Youden's J Statistic in the following formula:

○ *J = TPR - FPR*

where TPR = True Positive Rate and FPR=False Positive Rate.

J represents an array of differences between TPR and FPR of different points on the ROC curve. The index of the maximum value of J, argmax(J), was used as a criterion for selecting the cut-off that may represent the optimum threshold.

1. The optimum threshold was used to binarize the predicted probabilities into 0 and 1.
2. Links predicted from the model were verified across the ground truth links of G_𝜏+1_. The average performance metrics of the model were obtained by resampling the test set repeatedly for 100 times and testing 1000 samples in each iteration. The margin of error for 95% confidence intervals was also calculated.
