# Supplementary material for: Predicting Emerging Themes in Rapidly Expanding COVID-19 Literature With Unsupervised Word Embeddings and Machine Learning: Evidence-Based Study
Source: J Med Internet Res. 2022 Nov 2;24(11):e34067. doi: 10.2196/34067 (PMC9629347; doi:10.2196/34067)
Supplement: Multimedia Appendix 2 [file jmir_v24i11e34067_app2.docx]

**Multimedia Appendix 2.** Frequency of articles belonging to specific categories in the COVID-19 literature.

| **Category** | **Keywords** | **Article Count** |
| --- | --- | --- |
| Respiratory Disease | Pneumonia  Cardio  Rheumatic | 3055  1489  234 |
| Infectious Disease | HIV  Diarrhea  Tuberculosis | 615  75  197 |
| Maternal & Child Health | Pregnant  Maternal health  Child | 688  15  3287 |
| Cancer | Cancer | 2385 |
| Risk | Risk | 5400 |

The counts were based on the occurrence of the given keywords in the article’s title.
