# Supplementary material for: Predicting Emerging Themes in Rapidly Expanding COVID-19 Literature With Unsupervised Word Embeddings and Machine Learning: Evidence-Based Study
Source: J Med Internet Res. 2022 Nov 2;24(11):e34067. doi: 10.2196/34067 (PMC9629347; doi:10.2196/34067)
Supplement: Multimedia Appendix 3 [file jmir_v24i11e34067_app3.docx]

**Multimedia Appendix 3.** List of software and packages used for our study with their sources and identifiers for the reproducibility of this study.

| **Package/Library** | **Source** | **URL** |
| --- | --- | --- |
| Python (version 3.7.12) | python.org | <https://www.python.org/downloads/release/python-3712/> |
| nltk (version 3.2.5) | PyPI | <https://pypi.org/project/nltk/3.2.5/> |
| gensim (version 3.6.0) | PyPI | <https://pypi.org/project/gensim/3.6.0/> |
| scispacy (version 0.3.0) | PyPI | <https://pypi.org/project/scispacy/0.3.0/> |
| networkx (version 2.5) | PyPI | <https://pypi.org/project/networkx/2.5/> |
| infomap (version 1.6.0) | PyPI | <https://pypi.org/project/infomap/1.6.0/> |
| pandas (version 1.1.5) | PyPI | <https://pypi.org/project/pandas/1.1.5/> |
| numpy (version 1.19.5) | PyPI | <https://pypi.org/project/numpy/1.19.5/> |
| scikit-learn (version 0.22.2) | PyPI | <https://pypi.org/project/scikit-learn/0.22.2/> |
| xgboost (version 0.90) | PyPI | https://pypi.org/project/xgboost/0.90/ |
| statsmodels (version 0.10.2) | PyPI | <https://pypi.org/project/statsmodels/0.10.2/> |
