# Supplementary material for: Predicting Emerging Themes in Rapidly Expanding COVID-19 Literature With Unsupervised Word Embeddings and Machine Learning: Evidence-Based Study
Source: J Med Internet Res. 2022 Nov 2;24(11):e34067. doi: 10.2196/34067 (PMC9629347; doi:10.2196/34067)
Supplement: Multimedia Appendix 4 [file jmir_v24i11e34067_app4.docx]

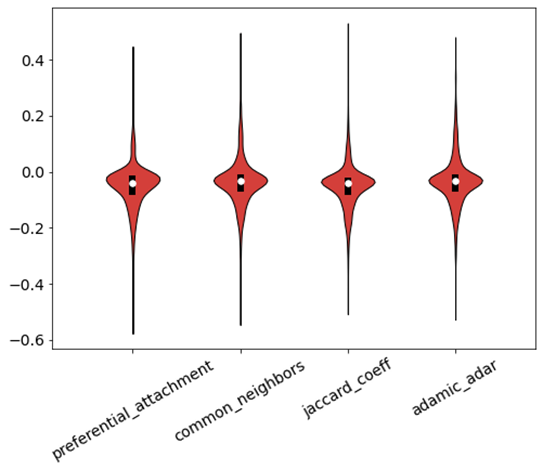


**Multimedia Appendix 4.** Distribution of errors in the prediction of proximity scores between node pairs (used as features in model training) for the month of June 2021. The white marker depicts the median and the black marker depicts the interquartile range. The mean of errors were found to be close to zero.
