# Supplementary material for: Predicting Emerging Themes in Rapidly Expanding COVID-19 Literature With Unsupervised Word Embeddings and Machine Learning: Evidence-Based Study
Source: J Med Internet Res. 2022 Nov 2;24(11):e34067. doi: 10.2196/34067 (PMC9629347; doi:10.2196/34067)
Supplement: Multimedia Appendix 5 [file jmir_v24i11e34067_app5.docx]

**Multimedia Appendix 5.** Models and respective parameters used for training.

| **Models** | **Parameters** |
| --- | --- |
| Word2Vec | vector_size=100, window=5, min_count=1, sg=1, epochs=5,  Default parameters from gensim.models.Word2Vec |
| Random Forest | n_estimators(trees)=100,  Default parameters from sklearn.ensemble.RandomForestClassifier |
| Support Vector Machine (SVM) | C(regularization parameter)=0.1,  Default parameters from sklearn.svm.SVC |
| XGBoost | n_estimators(trees)=500,  Default parameters from xgboost.XGBClassifier |
| AdaBoost | n_estimators(tress)=50, learning_rate=0.1,  Default parameters from sklearn.ensemble.AdaBoostClassifier |
