# Supplementary material for: Predicting Emerging Themes in Rapidly Expanding COVID-19 Literature With Unsupervised Word Embeddings and Machine Learning: Evidence-Based Study
Source: J Med Internet Res. 2022 Nov 2;24(11):e34067. doi: 10.2196/34067 (PMC9629347; doi:10.2196/34067)
Supplement: Multimedia Appendix 7 [file jmir_v24i11e34067_app7.docx]

**Multimedia Appendix 7.** The top 10 similar entities (diseases, conditions, or chemicals) with selected keywords (“vaccine,” “comorbidity,” “adverse effects,” “social,” and “psychological”) in descending order of cosine similarity calculated using the word embeddings generated from the Word2Vec model trained on the entire corpus.

| ***vaccine*** | ***comorbidity*** | ***adverse_effects*** | ***social*** | ***psychological*** |
| --- | --- | --- | --- | --- |
| mRNA-1273 | comorbid illnesses | clozapine-treated | social cohesion | distress |
| hesitancy | chronic pulmonary disease | maladaptive anxiety | psychological damage | social anxiety |
| Pfizer/ BioNTech | concomitant diseases | retinal adverse | sociability | burnout syndrome |
| BNT162b2 | chronic renal disease | Nosocomial co-infections | social connectedness | coronavirus stress |
| Moderna | cardiac disease | neuropsychiatric & neurodegenerative disease | social behavior | coping behaviors |
| MMR | CCI | sickle cell anemia | social stigma | posttraumatic stress symptoms |
| Ad26.COV2.S | chronic comorbidity | junction damage | social connectedness | COVID-19 anxiety |
| Oxford/ AstraZeneca | morbid obesity | cardiac ADRs | emotional labor | traumatization |
| AZD1222 | multi-morbidity | acute lower respiratory tract infection | family violence | disordered eating |
| ChAdOx1 | arterial hypertension | neurotic disorders | social anxiety | Depressive symptomatology |

The cosine similarity between entities has also been used as a feature for link prediction
