# Supplementary material for: Predicting Emerging Themes in Rapidly Expanding COVID-19 Literature With Unsupervised Word Embeddings and Machine Learning: Evidence-Based Study
Source: J Med Internet Res. 2022 Nov 2;24(11):e34067. doi: 10.2196/34067 (PMC9629347; doi:10.2196/34067)
Supplement: Multimedia Appendix 8 [file jmir_v24i11e34067_app8.docx]

**Multimedia Appendix 8.** Evaluation of the mean squared error between original and predicted proximity scores for the network of April 2021, May 2021, and June 2021.

| **Proximity Score** | **Mean Squared Error** | | |
| --- | --- | --- | --- |
|  | **April 2021** | **May 2021** | **June 2021** |
| Cosine Similarity | 0.089 | 0.052 | 0.034 |
| Jaccard Coefficient | 0.040 | 0.013 | 0.009 |
| Number of Common Neighbors | 0.050 | 0.014 | 0.009 |
| Preferential Attachment | 0.068 | 0.017 | 0.009 |
| Adamic-Adar Index | 0.044 | 0.013 | 0.009 |
