# Supplementary material for: Predicting Emerging Themes in Rapidly Expanding COVID-19 Literature With Unsupervised Word Embeddings and Machine Learning: Evidence-Based Study
Source: J Med Internet Res. 2022 Nov 2;24(11):e34067. doi: 10.2196/34067 (PMC9629347; doi:10.2196/34067)
Supplement: Multimedia Appendix 9 [file jmir_v24i11e34067_app9.docx]

**Multimedia Appendix 9.** Results of temporal link prediction between entities for the months of April 2021, May 2021, and June 2021, with a margin of error for 95% confidence intervals.

| **Model** | **April 2021** | | **May 2021** | | **June 2021** | |
| --- | --- | --- | --- | --- | --- | --- |
|  | **AUC ROC** | **Acc.** | **AUC ROC** | **Acc.** | **AUC ROC** | **Acc.** |
| **Random Forest** | 0.58 ± 0.0014 | 0.77 ± 0.0008 | 0.74 ± 0.0012 | 0.70 ± 0.0009 | 0.80 ± 0.0013 | 0.74 ± 0.0008 |
| **Support Vector Machine** | 0.51 **±** 0.0017 | 0.75 **±** 0.0009 | 0.79 **±** 0.0014 | 0.79 **±** 0.0009 | 0.85 **±** 0.0012 | 0.86 **±** 0.0007 |
| **ADABoost** | 0.52 ± 0.0016 | 0.74 **±** 0.001 | 0.81 ± 0.0011 | 0.77 **±** 0.0009 | 0.87 **±** 0.0009 | 0.83 **±** 0.0008 |
| **XGBoost** | 0.58 ± 0.0015 | 0.65 ± 0.0009 | 0.79 ± 0.0012 | 0.75 ± 0.0008 | 0.84 ± 0.0011 | 0.83 **±** 0.0008 |

The mean value of metrics has been recorded by testing the models on a resampled test set.
