# Supplementary material for: Predicting Emerging Themes in Rapidly Expanding COVID-19 Literature With Unsupervised Word Embeddings and Machine Learning: Evidence-Based Study
Source: J Med Internet Res. 2022 Nov 2;24(11):e34067. doi: 10.2196/34067 (PMC9629347; doi:10.2196/34067)
Supplement: Multimedia Appendix 10 [file jmir_v24i11e34067_app10.docx]

**Multimedia Appendix 10.** Welch *t* test results of the performance of algorithms for the test set of June 2021.

| **Model** | **RF** | **SVM** | **ADABoost** | **XGBoost** |
| --- | --- | --- | --- | --- |
| **RF** |  | *P*<.001 | *P*<.001 | *P*<.001 |
| **SVM** | *P*<.001 |  | P<.001 | P<.001 |
| **ADABoost** | *P*<.001 | *P*<.001 |  | P<.001 |
| **XGBoost** | *P*<.001 | *P*<.001 | *P*<.001 |  |

The mean AUC-ROC scores of all algorithms were found to be significantly different from each other with the Bonferroni-corrected significance level of α=.0083
