# Supplementary material for: Predicting Emerging Themes in Rapidly Expanding COVID-19 Literature With Unsupervised Word Embeddings and Machine Learning: Evidence-Based Study
Source: J Med Internet Res. 2022 Nov 2;24(11):e34067. doi: 10.2196/34067 (PMC9629347; doi:10.2196/34067)
Supplement: Multimedia Appendix 11 [file jmir_v24i11e34067_app11.docx]

**Multimedia Appendix 11.** Community detection results from the predicted and actual networks for June 2021.

| **Module ID** | **Top nodes in Predicted network** | **Module ID** | **Top nodes in Original network** |
| --- | --- | --- | --- |
| 1 | headache, lymphopenia, dyspnea, confusion, encephalitis, nausea | 1 | vomiting, nausea, headache, diarrhea, dyspnea, lymphopenia |
| 2 | fibrosis, coagulopathy, thrombotic, hypoxia, inflammation, delirium | 2 | Fibrosis, myocarditis, coagulopathy, hypoxemia, thromboembolic, shock, sepsis |
| 3 | comorbidity, asthma, COPD, hypertension, dementia, diabetes | 3 | Confusion, immunocompromised, traumatic, panic, scaly, cross-infection, cancer |
| 4 | traumatic, anxiety, depression, loneliness, burnout, insomnia | 4 | Comorbid, asthma, COPD, diabetes, hypertension, obesity |
| 5 | immunocompromised, chronic  diseases like tuberculosis | 5 | Anxiety, traumatic, depressive, depression, burnout, panic, insomnia, anger |

A subset of nodes from different modules have been shown for both predicted and actual networks
