# Supplementary material for: Predicting Emerging Themes in Rapidly Expanding COVID-19 Literature With Unsupervised Word Embeddings and Machine Learning: Evidence-Based Study
Source: J Med Internet Res. 2022 Nov 2;24(11):e34067. doi: 10.2196/34067 (PMC9629347; doi:10.2196/34067)
Supplement: Multimedia Appendix 12 [file jmir_v24i11e34067_app12.docx]

**Multimedia Appendix 12.** Results of community detection from the predicted subsequent network based on training data till June 2021.

| **Module ID** | **Theme** | **Subset of nodes from different modules** |
| --- | --- | --- |
| 1 | Adverse events (predisposing conditions and risk factors) | Myocarditis, coagulopathy, thromboembolic, hypoxemia, fibrosis, respiratory distress, immunocompromised |
| 2 | Symptoms (complications and symptoms of diseases) | Lymphopenia, dyspnoea, vomiting, diarrhea, dyspnea, nausea, headache, myalgia, anosmia |
| 3 | Respiratory illness | Respiratory infections, respiratory illness, respiratory infection, respiratory disease, coronavirus infection, mers |
| 4 | Neuro-psychiatric manifestation, Cross-infection | Confusion, psychiatric, cross-infection, trauma, pain, panic, labor, cross-infection, contagion |
| 5 | Psychological conditions | Traumatic, depression, depressive, anxiety, burnout, insomnia, psychological distress |

A subset of nodes was mentioned, which broadly signifies a theme for the given module.
