# Supplementary material for: Predicting Emerging Themes in Rapidly Expanding COVID-19 Literature With Unsupervised Word Embeddings and Machine Learning: Evidence-Based Study
Source: J Med Internet Res. 2022 Nov 2;24(11):e34067. doi: 10.2196/34067 (PMC9629347; doi:10.2196/34067)
Supplement: Multimedia Appendix 13 [file jmir_v24i11e34067_app13.docx]

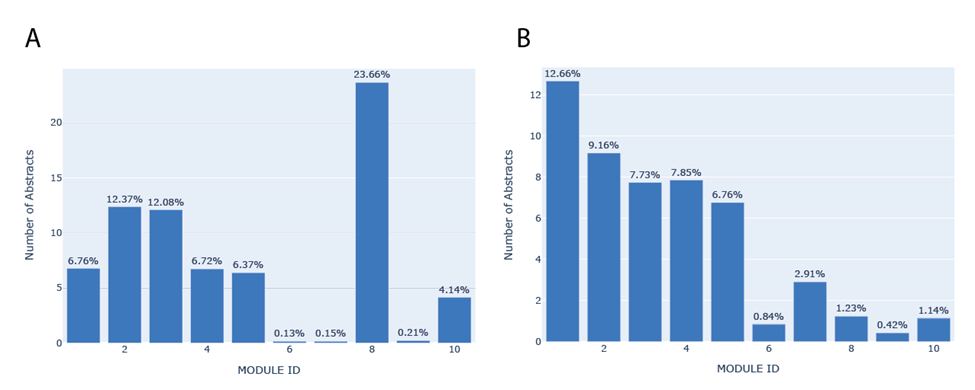


**Multimedia Appendix 13.** Percentage of abstracts of articles published in June 2021 mentioning diseases belonging to each module in the actual (A) and predicted (B) networks.
