# Supplementary material for: Predicting Emerging Themes in Rapidly Expanding COVID-19 Literature With Unsupervised Word Embeddings and Machine Learning: Evidence-Based Study
Source: J Med Internet Res. 2022 Nov 2;24(11):e34067. doi: 10.2196/34067 (PMC9629347; doi:10.2196/34067)
Supplement: Multimedia Appendix 14 [file jmir_v24i11e34067_app14.docx]

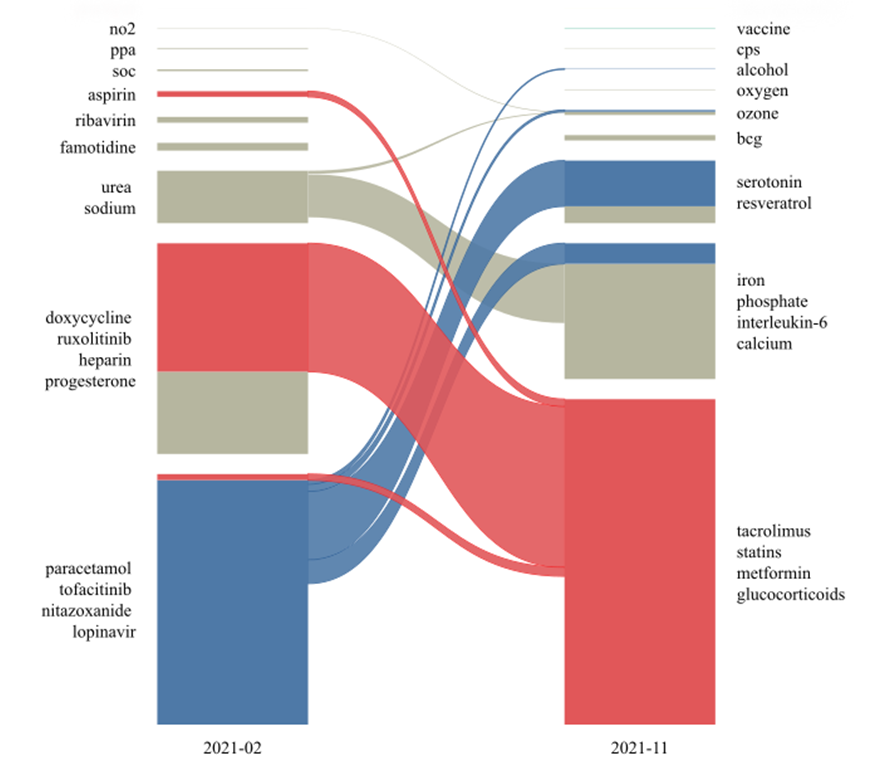


**Multimedia Appendix 14.** Alluvial diagram for tracking the trends of chemical entities from the networks of February 2020 to November 2021. Threshold used for assigning links between nodes was set at 70th percentile of cosine similarity between pairs of top-100 entities in respective months.
