# Supplementary material for: Predicting Emerging Themes in Rapidly Expanding COVID-19 Literature With Unsupervised Word Embeddings and Machine Learning: Evidence-Based Study
Source: J Med Internet Res. 2022 Nov 2;24(11):e34067. doi: 10.2196/34067 (PMC9629347; doi:10.2196/34067)
Supplement: Multimedia Appendix 15 [file jmir_v24i11e34067_app15.docx]

**A**

**
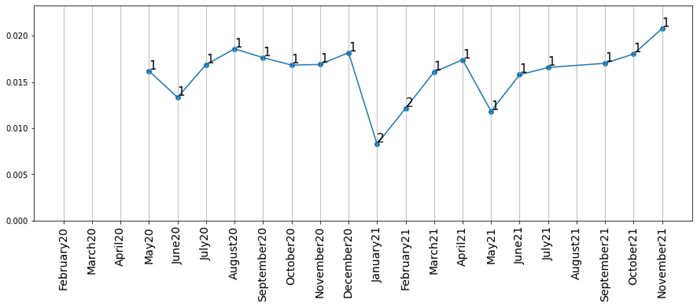
**

**B**

**
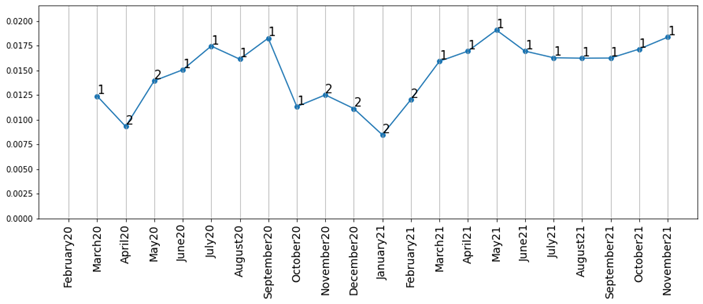
**

**C**

**
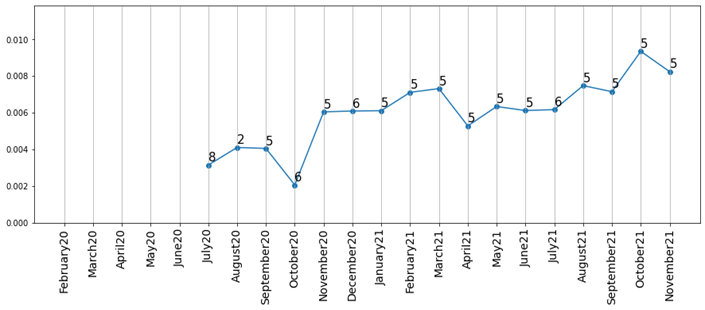
**

**D**


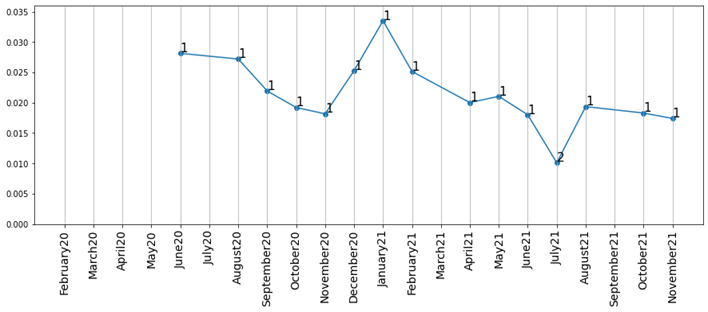


**Multimedia Appendix 15.** Temporal trends of the PageRank centrality of (A) “statins,” (B) “glucocorticoids,” (C) “depressive,” and (D) “thromboembolic.” The annotations denote the module/cluster index (lesser meaning more central).
